# Expression, Localization of SUMO-1, and Analyses of Potential SUMOylated Proteins in *Bubalus bubalis* Spermatozoa

**DOI:** 10.3389/fphys.2017.00354

**Published:** 2017-06-13

**Authors:** Rahim Dad Brohi, Li Wang, Najla Ben Hassine, Jing Cao, Hira Sajjad Talpur, Di Wu, Chun-Jie Huang, Zia-Ur Rehman, Dinesh Bhattarai, Li-Jun Huo

**Affiliations:** ^1^Key Laboratory of Agricultural Animal Genetics, Breeding and Reproduction, Education Ministry of China, College of Animal Science and Technology, Huazhong Agricultural UniversityWuhan, China; ^2^Department of Hubei Province's Engineering Research Center in Buffalo Breeding and ProductsWuhan, China; ^3^Department of Biology, University of Evry Val D'essonneEvry, France

**Keywords:** *Bubalus bubalis*, spermatozoa, protein, post translational modification, SUMO-1

## Abstract

Mature spermatozoa have highly condensed DNA that is essentially silent both transcriptionally and translationally. Therefore, post translational modifications are very important for regulating sperm motility, morphology, and for male fertility in general. Protein sumoylation was recently demonstrated in human and rodent spermatozoa, with potential consequences for sperm motility and DNA integrity. We examined the expression and localization of small ubiquitin-related modifier-1 (SUMO-1) in the sperm of water buffalo (*Bubalus bubalis*) using immunofluorescence analysis. We confirmed the expression of SUMO-1 in the acrosome. We further found that SUMO-1 was lost if the acrosome reaction was induced by calcium ionophore A23187. Proteins modified or conjugated by SUMO-1 in water buffalo sperm were pulled down and analyzed by mass spectrometry. Sixty proteins were identified, including proteins important for sperm morphology and motility, such as relaxin receptors and cytoskeletal proteins, including tubulin chains, actins, and dyneins. Forty-six proteins were predicted as potential sumoylation targets. The expression of SUMO-1 in the acrosome region of water buffalo sperm and the identification of potentially SUMOylated proteins important for sperm function implicates sumoylation as a crucial PTM related to sperm function.

## Introduction

Spermatozoa are highly regulated processes that consist of differentiated cells based on a head, mid-piece, and tail while the mammalian sperm head is composed of a nucleus and acrosome. The acrosome is a secretory vesicle, essential for sperm-egg fusion (Ramalho-Santos et al., 2002). The acrosome contains a variety of proteins required for sperm penetration through the zona pellucida (Kim et al., 2001) and fusion with the egg (Saxena et al., 2002). Bovine sperm contain hundreds of different proteins, and most of their functions are unknown, but it is believed that they are involved in different phases of fertilization. Thus, alterations in expression levels or post-translational modifications of specific proteins could alter sperm functions, potentially optimizing semen fertility. Mature spermatozoa have highly condensed DNA, which is silent at both the transcriptional and translational level (Baker et al., 2012; Rahman et al., 2013) or poorly capable of translation (Gur and Breitbart, 2006). Therefore, post translational modifications and their ability to regulate important sperm functions, including capacitation, motility, and the acrosome reaction, are all essential for oocyte penetration and are considered as the focus of studies of individual sperm proteomes (Pixton et al., 2004; Baker et al., 2005; Aitken and Baker, 2008; Oliva et al., 2009; Brohi and Huo, 2017). The literature reveals strong relationships between proteins and post-transcriptional modifications in spermatozoa which have a positive impact on fertility of men and mice, whereas, limited studies have been reported in buffalo bulls.

Sumoylation is a reversible post-translational protein modification, involved in the modulation of many biological systems (Yeh, 2009). SUMO is a highly-conserved 11 kDa post-translational protein modifier, which has a similar structure to ubiquitin and conjugates target proteins by a similar mechanism (Marchiani et al., 2011). However, rather than targeting proteins for degradation, sumoylation induces effects, such as altering protein localization or binding partners. An isopeptide bond is created between the glycine residues at the C-terminal end of SUMO with a lysine residue ε-amino group in the target protein. This dynamic process, which is catalyzed by activation, conjugation, and ligation enzymes (Geiss-Friedlander and Melchior, 2007), is transient and reversible (Marchiani et al., 2011); De-sumoylation is catalyzed by sentrin/SUMO-specific proteases (Yeh, 2009). SUMO has been implicated in the modulation of cellular functions as diverse as replication, the repair and recombination of DNA, the transcription of RNA, trafficking between the nucleus and cytoplasm, and protein stability (Seeler and Dejean, 2003; Girdwood et al., 2004; Yurchenko et al., 2006; Geiss-Friedlander and Melchior, 2007). Sumoylated proteins have been found in both the nucleus and the cytoplasm in germ and somatic cells and the both in mitochondria and plasma membrane (Yeh, 2009; Zunino et al., 2009).

Protein sumoylation during different stages of spermatogenesis was recently demonstrated in humans (Vigodner et al., 2006, 2013; Brown et al., 2008) and rodents (Rogers et al., 2004; Vigodner and Morris, 2005; Stanton et al., 2012), with localization of SUMO1 to chromatin and other cellular domains, both in germ cells, including spermatocytes and spermatids, and in somatic cells, including Leydig, Sertoli, and peri tubular myoepithelial cells. While the role of SUMO in spermatogenesis remains undefined, it is suggested to be involved in heterochromatin stability, DNA repair mechanisms and the regulation of gene expression (Rogers et al., 2004; Vigodner et al., 2006; Stanton et al., 2012; Marchiani et al., 2014). SUMO1 expression is demonstrated in a high percentage of live, mature human sperm (Marchiani et al., 2011), However, excessive levels of sumoylation may be linked to an abnormal sperm morphology and motility (Vigodner et al., 2013; Marchiani et al., 2014) and to sperm DNA fragmentation (Marchiani et al., 2014). The localization of SUMO in sperm and its target proteins defined its promising role in different circumstances. Targets that have been identified in mature human sperm include the mitochondrial protein DRP1, the microtubule-organizing protein RanGAP1 and topoisomerase IIa, which is involved in chromatin remodeling (Marchiani et al., 2014). More studies should be conducted on the proteomic profile of sperm, particularly post-transcriptional protein modifications such as SUMOylation, which might give new insights to resolving infertility issues. The objective of this study was to evaluate SUMO1 expression and localization in ejaculated spermatozoa from Buffalo bull and identify protein targets of sumoylation in this model.

## Materials and methods

### Ethics statement (for mice)

This current study was approved by the Research Center of Experimental Animals (Approval ID: JkXK (Hb) 200980006) of the Ethical Committee. In this present study, Kunming mice were obtained from the local Central Animal Laboratory and were kept in the experimental animal center at the University at a temperature of 22°C with access to water and food *ad libitum*. In this study, all of the experimental procedures were performed according the guidelines of the Committee of the Animal Research Institute of the University.

### Buffalo bull semen

Frozen buffalo bull semen was purchased from a local Breeding Bull station of Hubei Province, Chinawere used for the present study. The Bull spermatozoa were frozen in liquid nitrogen and, for the experiments, were thawed out in a water bath at 37°C and were separated by percoll density gradient centrifugation, as described previously (Marchiani et al., 2014).

### Mouse sperm

Kunming mice sperm were obtained from the cauda epididymis by mincing the epididymis tissue in PBS. This washed mouse sperm were attached to poly-lysine coated slides for further use in experiments.

### Chemicals and solutions

The polyclonal anti-SUMO-1 antibody (catalog no: sc-9060) was purchased from Santa Cruz biotechnology, Inc. (Texas, U.S.A), the FITC-PNA staining kit was from GENMED SCIENTIFIC (Shanghai, China) and all other reagents were purchased from Sigma-Aldrich Chemical Co., USA, unless stated otherwise.

### Coomassie brilliant blue staining of acrosome of bull sperm

Assessments of buffalo bull acrosomal status were conducted essentially as previously described (Hutt et al., 2005). Briefly, fixed spermatozoa were collected by centrifugation at 800 × g for 5 min and washed two times in 100 mM ammonium acetate, pH 9.0 (1 ml). The spermatozoa were subsequently resuspended in 0.5 ml of 100 mM ammonium acetate, pH 9.0, the sample was smeared and allowed to air-dry on glass slides. After air drying, the slides were rinsed with tap water, methanol, and water for 5 min each. The sperm were stained for 10 min at RT with Coomassie Brilliant Blue G-250 (50% methanol and 10% acetic acid). The slides were washed three times with dH2O, air-dried, and mounted in glycerol (30% in PBS) before imaging with a Nikon inverted fluorescence microscope to analyze acrosomal staining.

### FITC-PNA staining of acrosome of bull sperm

The sperm acrosome and nuclei were assessed using FITC-PNA and 4, 6-diamino-2-phenylindole (DAPI) staining, respectively. The sperm acrosome status was assessed by applying FITC-PNA staining kit (GENMED SCIENTIFIC, Shanghai, China), which is specific for sperm acrosome staining. Briefly, after incubation at 37°C for 30 min, the bull spermatozoa specimen was centrifuged for 5 min at 600 × g, the sample was collected and resuspended in a preservative solution at a final concentration of 2 × 10^7^ sperms cell/ml. A 20 μl sperm sample was smeared over a microscope glass slide at room temperature and was fixed using an ice cold fixing solution for 60 s. After drying, 200 μl of FITC-PNA stain (25 mg/ml) were spread gently over the smear and the slide was incubated in a dark chamber for 20 min at room temperature. The prepared glass slide was further washed with 200 μl PBS or cleaning solution to displace surplus stain. The sperm acrosome status was assessed and photographs were taken using an *epifluorescence* microscope (Olympus) at wavelengths of 480 and 530 nm.

### Immunofluorescence staining of SUMO-1 in bull sperm

Sperm cells, recovered from the Percoll gradient, were washed in PBS. The washed buffalo bull sperm were attached to slides and fixed in 4% formaldehyde (Beyotimes, China) for 20 min at RT and the slides were rinsed three times in PBS. The fixed sperm cells were treated for 10 min with 0.3% Triton x100, and the slides were pre-blocked for 60 min with 5% BSA placed at 4°C. The sperm cells were washed with PBS and were incubated with anti SUMO-1 antibody (1:250 in PBS/1% BSA) overnight at 4°C in a dark chamber. The cells were washed three times with PBS and were then incubated with CY-3 labeled secondary antibody (Boster Co., China) at a 1:150 dilution for 60 min at 37°C in a dark room. The cells were then washed 3 × 5 min with PBS before nuclear staining for 5 min with (DAPI, 1:5,000 in PBS) in a dark room. The specimens were washed, and the glass slides were mounted in DABCO. The images were finally analyzed with a Nikon inverted fluorescence microscope with 60 and 100x objective lenses and DAPI, FITC, and CY-3 filter settings. Minimally, 50 cells were analyzed per slide. In order to determine the co-localization of SUMO-1 with acrosome in bull sperm, after immunostaining the sperm with SUMO-1, the slides were further incubated with FITC-PNA as described above.

### Protein extraction of buffalo sperm and co-immunoprecipitation

Protein extraction was performed as previously described (Sang et al., 2011; Kumar et al., 2014). Briefly, after the final wash in PBS, the sperm pellets were resuspended in 400 μl 2x Laemmli buffer [126 mM Tris/HCl, 20% glycerol, 4% SDS, 10 mM PMSF, protease inhibitor cocktail (10 mg/ml; Santa Cruz Biotechnology, CA, USA)], containing isopeptidase inhibitor NEM (Sigma), to inhibit SUMO cleavage from modified proteins. The samples were heated to 100°C (5 min), placed on ice (5 min) and centrifuged at 12,000 × g for 2 min at 4°C before collecting the supernatants. The protein concentration was determined by the BCA assay with a BSA standard (Pierce, Rockford, USA). The samples were stored at −20°C for later use.

Anti-SUMO-1 antibodies and protein A+G Agarose (Beyotimes, China) were used for the Co-Immunoprecipitation according a manufacturer's instructions. Briefly, the sperm protein extracts were incubated with anti-SUMO-1 polyclonal antibody over-night at 4°C on constant shaker. The next day, 40 μl Protein A+G Agarose were added, and the samples were incubated at 4°C with constant shaking. The resin was washed five times with ice cold PBS, and the pellets were resuspended in 2x SDS loading buffer and were heated for 5 min at 100°C before SDS-PAGE electrophoresis for western blotting or mass spectrometry.

### Western blot analysis

After measuring the protein concentration (determined by BCA assay), the sperm lysates were mixed with 2x SDS loading buffer and were loaded over 10% polyacrylamide gels. After SDS-PAGE, the proteins were transferred to PVDF membranes (Millipore Corp., Bedford, MA). The membranes were blocked with 5% skim milk (Sigma-Aldrich), in TBST [10 mM Tris (pH 7.5), 150 mM NaCl and 0.05% Tween 20], and were then incubated with the polyclonal rabbit anti-SUMO-1 IgG (1:500) diluted in blocking buffer overnight at 4°C. Afterward the membrane was rinsed three times in TBST and was then incubated with HRP conjugated anti-rabbit IgG antibody in TBST for 60 min at room temperature and was finally rinsed three times in TBST. Detection of the bands was accomplished with a BM-enhanced chemiluminescence system (Amersham Biosciences, Piscataway, NJ).

### Spermatozoa capacitation and acrosome reaction *in vitro*

The acrosome reactions were stimulated as previously described (Kumar et al., 2014). In brief, the sperm were capacitated *in vitro* by thawing of frozen semen at 37°C for 40 s and centrifugation at 1,500 rpm for 25 min. The pellets were reconstituted in 2 ml of a modified Tyrode's Hepes-buffered (Sp-TALPH) washing medium and centrifuged twice, at 1,500 rpm for 10 min. After centrifugation, the pellets were washed with a modified Tyrode's bicarbonate-buffered (Sp-TALP). The pellets were re-suspended in Sp-TALP containing concentration of 25 × 10^6^ spermatozoa in a final volume of 0.5 ml in an Eppendorf tube and capacitation was induced by the presence of 10 μg/ml heparin with an incubation for 4 h at the 38.5°C in 5% CO_2_. The capacitated spermatozoa were treated for 30 min with 1 mM calcium ionophore A23187 (Sigma Chemical Co.). The acrosome reaction was confirmed by FITC-PNA staining.

### Mass spectrometry analysis of SUMO-1 targeted proteins in buffalo sperm

Protein digestion was conducted in accordance with the FASP method, essentially as earlier reported (Wisniewski et al., 2009). Briefly, the protein pellet (~30 μg) was solubilized in 30 μl SDT buffer (4% SDS, 100 mM DTT, 150 mM Tris-HCL pH 8.0) at 90°C for 5 min. The detergent, DTT and other low molecular weight components were removed using 200 μl UA buffer (8 M Urea, 150 mM Tris Hcl pH 8.0) by repeated ultrafiltration (Microcon units, 30 kD). Then 100 μl of 0.05 M iodacetamide in UA buffer was added to block the reduced cysteine residues and the samples were incubated in the dark for 20 min. The filters were rinsed with 100 μl of UA buffer three times and then with 100 μl of 25 mM NH_4_HCO_3_ twice. Finally, the protein suspension was digested with 2 μg trypsin (Promega) in 40 μl 25 mM NH_4_HCO_3_ overnight at 37°C, and the ensuing peptides were collected. The mass spectrometry investigations were conducted in a Q Exactive MS that was linked to easy nLC (Proxeon Biosystem, now Thermo Fisher Scientific). Six microliters of each fraction was injected for the nanoLC-MS/MS investigation. A peptide admixture of 5 μg was loaded onto a C18 reverse phase column (Thermo scientific easy column, 10 cm long, 75 μm inner diameter, 3 μm resin) into buffer A (Formic acid 0.1%) and was separated by a linear gradient of buffer B (acetonitrile 80% and Formic acid 0.1%) at a 250 nl/min flow rate controlled by Inelli Flow technology over 140 min. The mass spectrometry data were procured utilizing a data dependent top10 procedure energetically selecting the most abundant precursor ions from the survey, scan (300–1800 m/z) for HCD fragmentation. The fortitude of the target value was based pAGC. The dynamical elimination period was 1 min. The investigating scans were acquired by data determination in 70,000 at m/z 200, and the resolutions for the HCD spectra were fixed in 17,500 at m/z 200. The normalized collision energy was 30 eV under a fill ratio that specializes the minimum percentage of target value probable to be attained at the maximum fill time, which was definite as 0.1%. The equipment was run in an enabled mode of peptide identification.

MS/MS spectrometry was investigated utilizing a MASCOT engine (Matrix Science, London, UK; version 2.2) with a non-redundant International Protein Index Arabidopsis sequence database v3.85, declared and released from the European Bioinformatics Institute (http://www.ebi.ac.uk/). For the identification of proteins, the following adoptions were utilized: peptide mass tolerance = 20 ppm; MS/MS tolerance = 0.1 Da; Enzyme = Trypsin; Missed cleavage = 2; fixed modification: Carbamidomethyl (C), and Variable modification: Oxidation (M). The entire described data were established on a 99% assurance for protein discovery as resolute by a false discovery rate (FDR) of ≤ 1%.

### Bioinformatics analysis

Bio-analysis was performed under Ubuntu 16.04 LTS Docker image (Hung et al., 2016) within a custom Python (Python Software Foundation, 2015) scripts using the packages biopython, pyexcel, csv, collection, panda, and matplotlib and R v3.3.1 (R Core Team, 2016) scripts within the ggplot2 and plotrix libraries. Based on the MS/MS identified proteins listed (Table S1), sequences matching with *Bubalus bubalis* genome (41,499, protein, assembly UMD_CASPUR_WB_2.0) were searched against the NCBI databases (September-2016, https://www.ncbi.nlm.nih.gov) (Geer et al., 2010). The Prediction of Sub Cellular Location (SCL) was performed using two packages for prediction in eukaryotic and animal proteins with single or multiple sites as follows: (I) iLoc-Euk (http://www.jci-bioinfo.cn/iLoc-Euk) (Chou et al., 2011) and (II) iLoc-Animal (http://www.jci-bioinfo.cn/iLoc-Animal) (Lin et al., 2013). The protein sequences (in fasta format) were sent via user interfaces of the dedicated package's web servers. For further understanding of the *B. bubalis* sperm ejaculated proteome profile, we classified proteins of interest following three strategies: (I) COG (Clusters of Orthologous Group) classification; (II) Protein function classification; and (III) GO Slim (Huntley et al., 2014) classification. The first strategy, the COG analysis, was completed with EggNOG v4.5 (a database of Orthologous Groups and functional annotation covering prokaryotic and eukaryotic species, http://eggnogdb.embl.de) (Huerta-Cepas et al., 2016). The protein sequences were submitted to the web server API. Sequences longer than 800aa were processed with HMMER v3.1b2 (A software suite widely used for analysis of homologous protein and nucleotide sequences with high speed and sensitivity, http://hmmer.org) (Jiang and Ganesan, 2016). HMMER was used for homology sequences, searching within the EggNOG mammalian database annotation (maNOG.hmm). The results were filtered based on score and E-value significance.

The second strategy was the protein function classification and Gene Ontology (GO), based. Analysis was processed by PANTHER v11 (Protein Analysis Through Evolutionary Relationships, http://pantherdb.org) (Mi et al., 2016) with a statistical overrepresentation test and default setting criteria. The third strategy consisted of GO Slim (Huntley et al., 2014) classification. The GO terms reported by PANTHER were classified by the web server tool CateGOrizer v3.218 (May06-2010, A Web-Based Program to Batch Analyze Gene Ontology Classification Categories: http://www.animalgenome.org) (Von Mering et al., 2003).

Conscious about the crucial role of protein-protein interaction (PPI) in spermatozoa process, additional information about PPIs, pathways and COG classification were collected by STRING v10. 0. (A Database of predicted functional associations between proteins, http://string-db.org) (Hu et al., 2008).

The tracking of the PTMs in the protein list previously identified by MS/MS focused on SUMOylation, which one of the most important PTMs affecting the sperm proteome of water buffalo. In order to identify SUMOylated modified protein and SUMO-1 targets, we used several tools for predicting SUMOylation sites, motifs and interactions. Thus, the protein sequences were submitted to (i) GPS-SUMO-2.0 (a tool for the prediction of sumoylation sites and SUMO-interaction motifs, http://sumosp.biocuckoo.org) (Zhao et al., 2014) and (ii) JASSA (a comprehensive tool for prediction of SUMOylation sites and SIMs, http://www.jassa.fr) (Beauclair et al., 2015). The peptide sequence visualization was performed by Seq2Logo (Thomsen and Nielsen, 2012) using the following Parameters: PSSM_LOG; Halfbits and 800 × 800.

## Results

### Coomassie brilliant blue and FITC-PNA staining of acrosome in water buffalo sperm

**Figure 1:**
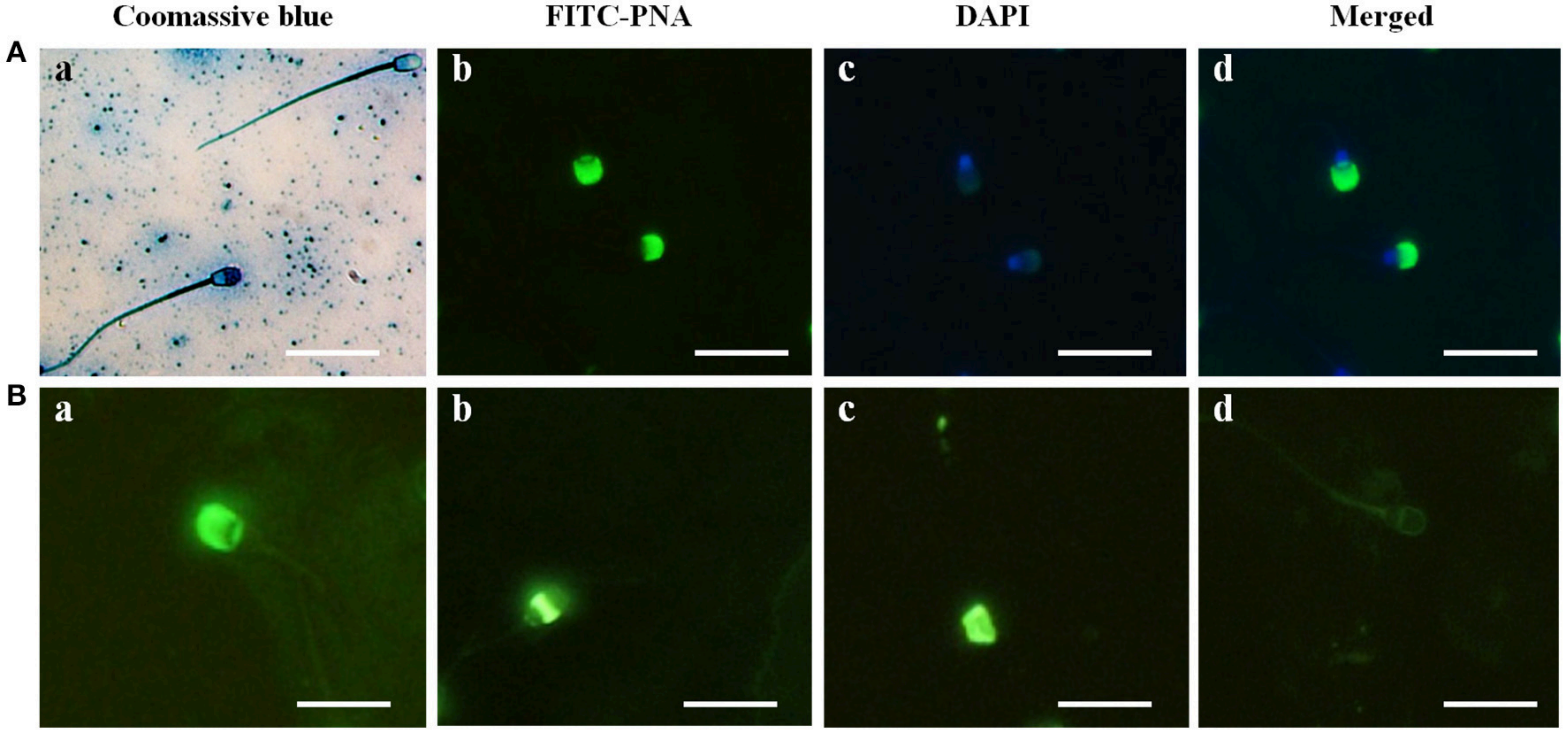
Acrosome identification in buffalo bull sperm and four categories of Buffalo bull sperm acrosome status. **(A)** (Image a) Coomassie Brilliant Blue staining of sperm with an intact and reacted acrosome, (image b) FITC-PNA stained, (image c) DAPI-stained, (image d) DAPI+FITC-PNA- stained images. **(B)** (Image a) intact acrosome, (image b) equatorial acrosome region, (image c) post acrosomal region, (image d) reacted acrosome. Scale bar, 20 μm; Magnification, 1,000x.

In the sperm from Buffalo bull, the acrosome was clearly identified by intense blue staining with Coomassie Brilliant Blue (Figure 1A image a), and the acrosome reaction was confirmed by loss of the Coomassie staining in the acrosome region of the sperm (Figure 1A image a). The evaluation of nuclear and acrosome morphology of the frozen-thawed bull spermatozoa was assessed with DAPI and FITC-PNA staining, respectively, indicating that the acrosome was enveloped with green fluorescence of FITC in the bull spermatozoa (Figure 1A images b–d). The acrosome status of the bull spermatozoa was evaluated through FITC-PNA (Figure 1B) and was classified within four categories. In category I (Figure 1B image a), the spermatozoa showed a strong, bright fluorescence in the acrosomal cap-like structure, resulting from the PNA binding, demonstrating an intact acrosome. In category II (Figure 1B image b), the fluorescence of the acrosomal cap was disrupted or there was fluorescence in the equatorial plane. Category III (Figure 1B image c) displayed fluorescence in the post acrosomal region of the sperm, and category IV (Figure 1B image d) spermatozoa displayed no fluorescence in the acrosome area, indicating a reacted acrosome. The results demonstrated that Coomassie Brilliant Blue staining and the FITC-PNA staining of sperm could be used to evaluate the acrosome status of water buffalo sperm.

### Immunocytochemical localization of SUMO-1 in bull spermatozoa

**Figure 2:**
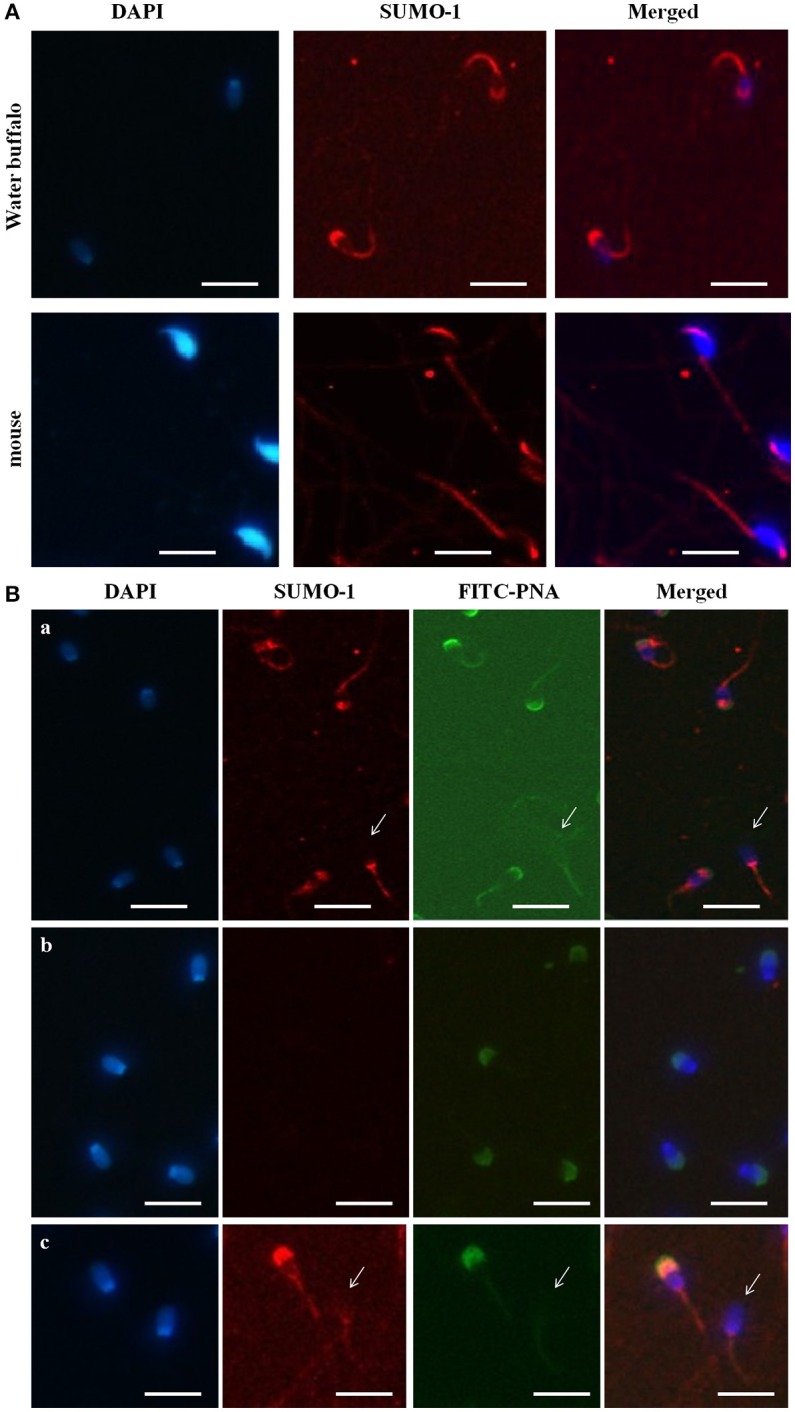
Localization of SUMO-1 in the acrosome of Buffalo bull and mouse spermatozoa. **(A)** DAPI, for nuclear staining (blue signal), SUMO-1, Anti-SUMO1 antibody followed by a CY3-conjugated secondary antibody (Red fluorescence), Merged, DAPI nuclear staining plus anti-SUMO1 antibody. **(B)** SUMO1 co-localization and expression in buffalo bull sperm. Collected spermatozoa were stained with SUMO1 antibody pursued by incubation with Cy-3 conjugated goat anti rabbit IgG (color red). The specimen were then restained with 4, 6-diamino-2-phenylindole (DAPI, 1:5,000 in PBS) (blue color) and FITC-PNA (flurescein isothiocyanate-conjugated peanut agglutinin) (green color). In (panel a) the superposed images shown in the spermatozoa were stained with SUMO-1 antibody followed by incubation with Cy-3 conjugated goat anti rabbit IgG. (panel b) the superposed images are shown in negative samples as control. (panel c) the images show both an intact and reacted acrosome, and the disappearance of SUMO-1 in the acrosome-reacted buffalo bull spermatozoa. Note that both SUMO-1 and FITC-PNA disappeared in acrosome region of the acrosome-reacted spermatozoa. The images were observed using a Nikon inverted fluorescence microscope. The scale bar is 100 μm.

The immunofluorescence staining demonstrated the mid piece, neck, head, and acrosome localization of SUMO-1 in bull spermatozoa (Figure 2A). At high magnification on the optimal immune stained samples, the SUMO-1 signal was observed in the acrosome region of sperm heads (Figure 2A). The SUMO-1 staining was not observed in the controls incubated with rabbit IgG instead of anti- SUMO-1 antibody. Additionally, signal specificity was confirmed using a secondary antibody alone under the same fluorescence intensity condition. For comparison, mouse epididymal spermatozoa were also immune-stained within anti SUMO1 antibodies. The SUMO-1 expression was similar to the bull spermatozoa (Figure 2A).

### Co-localization of SUMO-1 in bull spermatozoa

In order to further reveal the relationship of SUMO-1 localization with the acrosome of bull sperm, FITC-PNA staining of bull sperm was also used to co-localize the expression SUMO-1 and the acrosome in bull sperm (Figure 2B). The results demonstrated that SUMO-1 immunosignal was observed in the mid piece, neck, head, and acrosome region of the sperm head, overlapping with the PNA lectin acrosomal labeling (Figure 2B panel a). The immunosignal in the bull spermatozoa was not observed in the control sperm stained with rabbit IgG. The use of secondary antibody alone confirmed the specificity of the signal (Figure 2B panel b).

We further examined whether acrosome-localized SUMO-1 is released from buffalo bull spermatozoa during the acrosome reaction. Capacitated spermatozoa were induced to undergo acrosome reaction by Ca^2+^ ionophore A23187 and the acrosome status was monitored by FITC-PNA staining. The sperm showed FITC-PNA and SUMO-1 staining on an acrosomal region in spermatozoa with an intact acrosome (Figure 2B panel c, left), and this disappeared in the acrosome-reacted spermatozoa (Figure 2B panel c, right and also see Figure 2B panel a, arrow indicated sperm without an acrosome).

### Expression of SUMO-1 in capacitated and acrosome reacted spermatozoa of water buffalo

**Figure 3:**
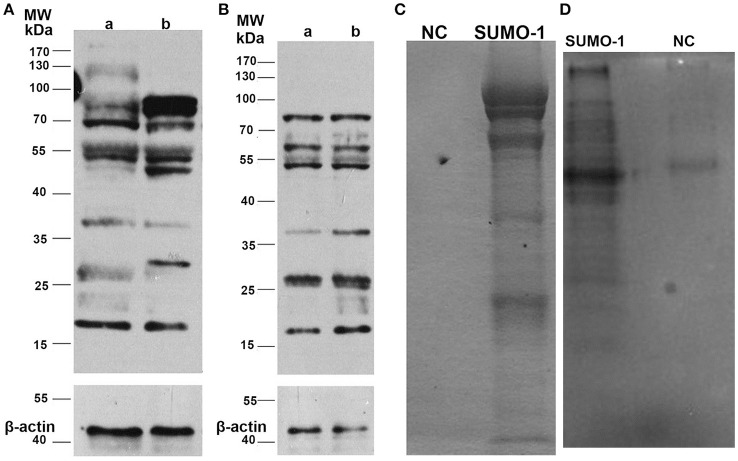
Expression of SUMO-1 in water buffalo spermatozoa. **(A)** Western blot analysis of SUMO-1 expression in water buffalo spermatozoa after *in vitro* capacitation, showing capacitation-induced changes in SUMO-1-modifications in sperm (lane b) compared to non-capacitated sperm (lane a). **(B)** Western blot analysis of acrosome reacted (lane a) vs. acrosome non-reacted (lane b) water buffalo spermatozoa. **(C)** Buffalo bull sperm proteins were immunoprecipatated with SUMO-1 antibody, and then run for SDS-PAGE and stained with coomassive brilliant blue. For the negative control (NC). **(D)** SUMO-1 conjugates were pull down from water buffalo sperm lysates by a SUMO-1 antibody and were analyzed by western blot to detect SUMO-1 expression.

To assess the expression profile of SUMO-1 between capacitated sperm and non-capacitated sperm in water buffalo after *in vitro* capacitation, protein samples extracted from the bull spermatozoa were run analyzed by western blot. After *in vitro* capacitation, SUMO-1-modifications were observed compared to the non-capacitated sperm (Figure 3A). After capacitation, bands at ~130, 37, and 27 kDa showed a lower intensity, a band at 30 kDa appears and bands at 85 and 50 kDa are enhanced (Figure 3A). Furthermore, after capacitation, the spermatozoa were induced to undergo the acrosome reaction by calcium ionophore A23187, and the expression of SUMO-1 in acrosome reacted sperm was also detected and was compared with the acrosome intact sperm (Figure 3B). There were subtle changes in the SUMO-1 expression profile after the acrosome reaction was induced. The acrosome reaction caused the band at 37 kDa to decrease and a smear of bands below 25 kDa disappeared (Figure 3B).

### SUMO-1 conjugations and mass spectrometry analysis

The SUMO-1 conjugates in water buffalo spermatozoa were first pulled down by Co-Immunoprecipitation using protein A+G agarose and the SUMO-1 antibody, and this was confirmed by Coomassive Brilliant Blue staining of the SDS-PAGE and by a Western blot analysis (Figures 3C,D). The results demonstrated that specific protein bands in the sample with SUMO-1 pull down, but not in the negative control sample (normal rabbit IgG) (Figures 3C,D), indicating that SUMO-1 specific conjugates were pulled down. These bands from the Coomassie blue stained gel (Figure 3C) were analyzed by mass spectrometry, resulting in the identification of 60 proteins that were unique to the SUMO-1 antibody sample compared to the negative control (Table S1). Ten of these proteins were identified in previous studies as being sumoylated, and these are indicated by an asterisk in Table S1.

The identified proteins have multiple functions in germ cells and sperm, including proteins associated with sperm motility, such as tubulin alpha-1C chain andalpha-1B chain, relaxin receptors RXFP4 relaxin (RLN)/insulin like family peptide receptor 4, RLN-3/INSL7 receptor 2, and LOC102283812 RLN-3 receptor 1-like, alpha and gamma actins ACTC1 and ACTG2.

### Bioinformatics findings

#### Database searches

In the present work, the 60 proteins identified by MS/MS were submitted to NCBI (September-2016, https://www.ncbi.nlm.nih.gov) in order to get their correspondent (fasta) sequences. We found 110 proteins matched with *B. bubalis* annotated protein sequences, and 92% of them were predicted. Many isoforms (65 proteins with at least two matches) were present in the list. The lack of publication and confirmation for most of the *B. bubalis* sequences prompted us to keep all of isoforms for the rest of the analyses.

#### Sub-cellular localization analysis

**Figure 4:**
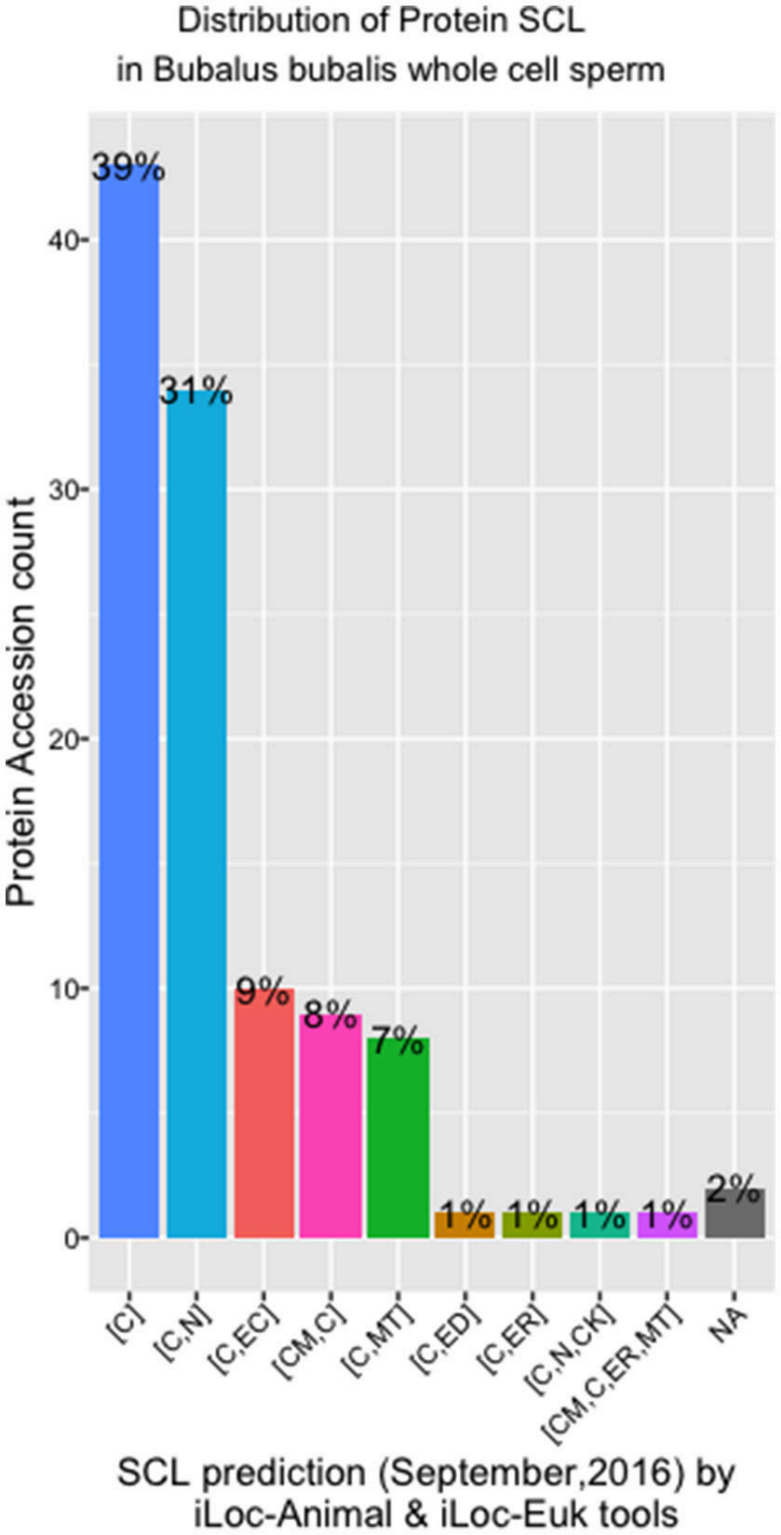
SCL in whole cell sperm in *Bubalus bubalis*. Plot describing the distribution of protein Sub-cellular localization [SCL] predicted by iLoc-Animal and iLoc-Euk tools. [C]: cytoplasm; [C,N]: cytoplasm and nculeus; [C,EC]: cytoplasm and extracellular; [CM,C]: cell membrane and cytoplasm; [C,MT]: cytoplasm and mitochondrion; [C,ED]: cytoplasm and endosome; [C,ER]: cytoplasm and endoplasmic reticulum; [C,N,CK]: cytoplasm, nucleus, cytoskeleton; [C,N,ER,MT]: cell membrane, cytoplasm, endoplasmic reticulum, and mitochondrion; [NA]: No Available results for those sequences.

There were eight different SCL predicted by both the iLoc-Animal (Lin et al., 2013) and iLoc-Euk (Chou et al., 2011) tools. We show that 39% of the whole cell sperm proteins are located only in the cytoplasm (Figure 4), and 31% are predicted in both the cytoplasm and nucleus. Tubulin alpha-1A chain-like [XP_006040217.1] is the only protein of the list present in the cytoskeleton in addition of his presence in the cytoplasm and nucleus. Two proteins: *polyribonucleotide nucleotidyltransferase1* PNPT1 (6 isoforms) and *metalloendopeptidase* OMA1 (2 isoforms) known for their crucial role in the mitochondria, were predicted in both cytoplasm and mitochondrion (Table S2). We observed that *cadherin EGF LAG seven-pass G-type receptor 2* [XP_006052222.1] and *ADP-ribosyltransferase4* (ART4) [ABO72473.1] were located in the endoplasmic reticulum. *Dombrock blood group protein* (ART4) was predicted as known in humans, in the cell membrane, cytoplasm and mitochondrion also. In addition, we noted that *phosphatidylinositol 3-ki0se catalytic subunit type 3-like* [XP_006057019.1] is implicate in many pathways trafficking and was predicted in the cytoplasm and endosomes, which is known in humans.

#### Protein classification

**Figure 5:**
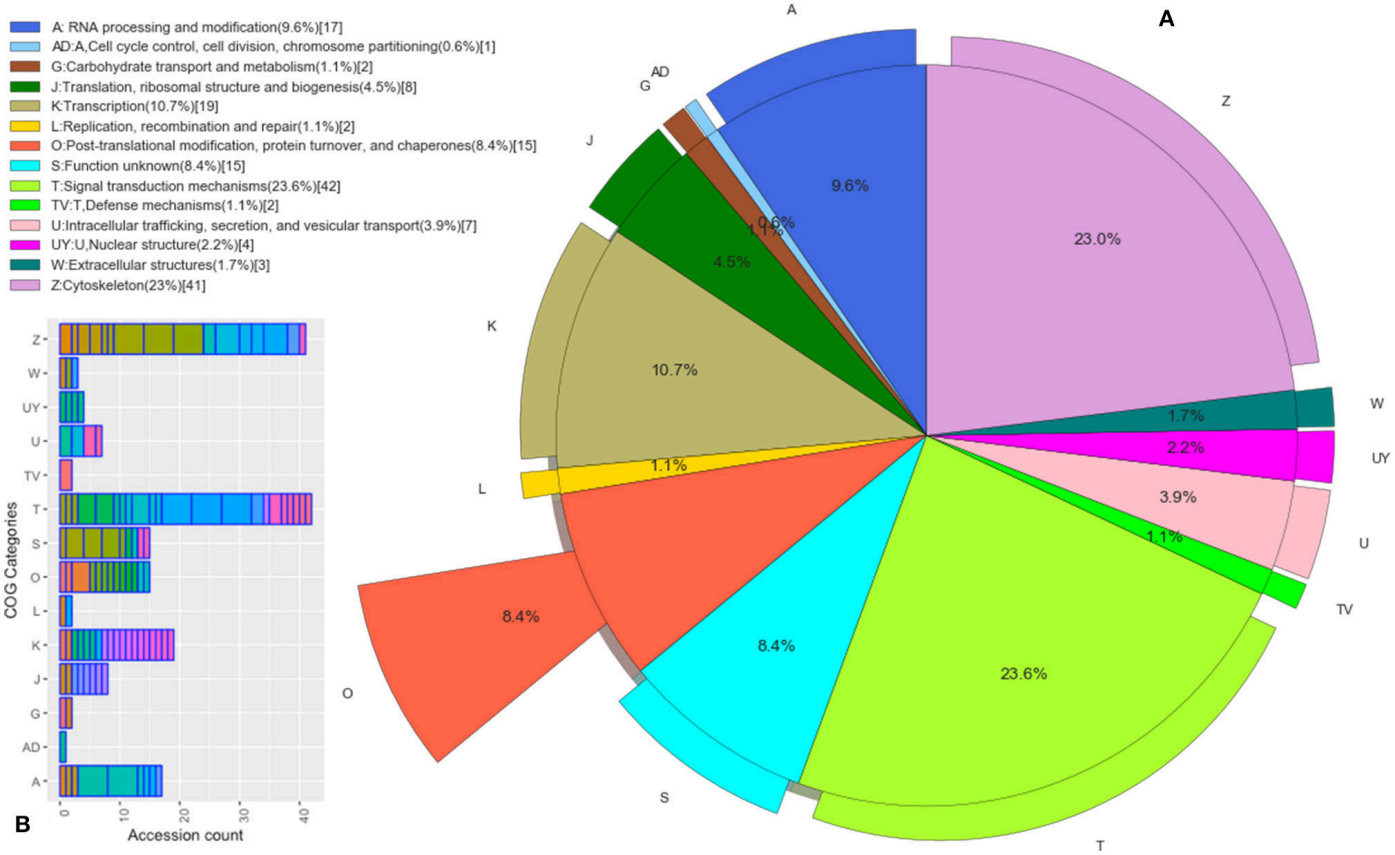
Functional Protein Classification (COGs). **(A)** Distribution of proteins count in the COGs categories. Colored boxes correspond to the unique COGs subcategories (EggNOG v4.5 terms). **(B)** The pie shown each COG category percentage.

**Figure 6:**
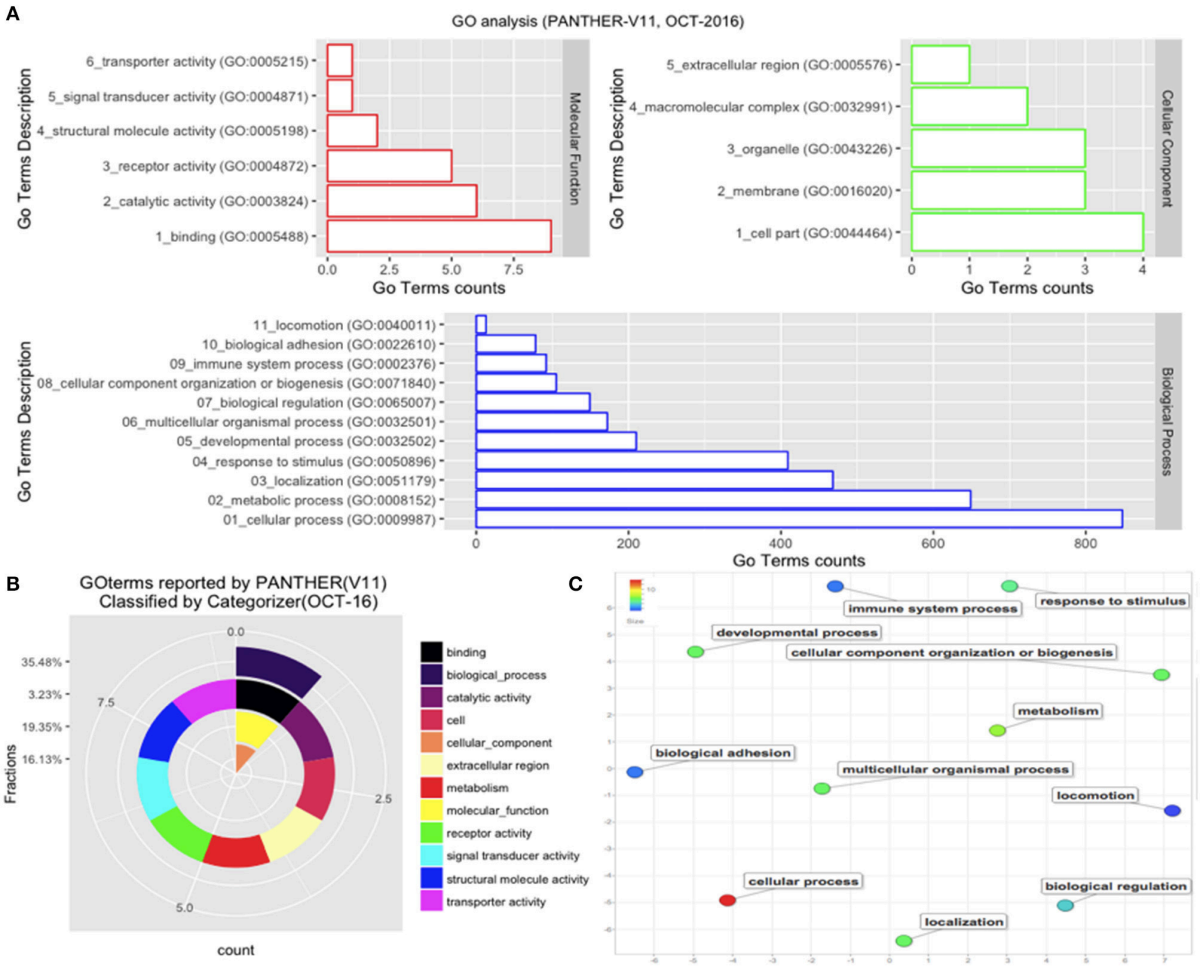
GO analysis. The GO analysis was performed with PANTHER (V11). **(A)** Details of GO terms distribution. **(B)** The terms were reclassified using Categorizer [http://animalgenome.org]. This represents the mapping of 22 GO terms to 127 of the “GO Slim” ancestor terms by single count. **(C)** Plot of GO terms details. Further analysis of GO terms was conducted by Rivgo [http://revigo.irb.hr].

Those *B. bubalis* proteins previously identified were functionally classified through to GO and COG strategies. The EggNOG (Huerta-Cepas et al., 2016) results (270 match) were filtered based on the score and E-value criteria (Score>100, E-value <1E-180). We successfully affected 178 filtered entries in 14 COG categories (Figure 5). The COG analysis reveals that 8% of those proteins belong to (O: Post-translational modification, protein turnover, and chaperones), 23% to (Z: Cytoskeleton), 23% to (T: Signal transduction mechanisms), and 10% to (K: Transcription) COGs categories. Also, we subjected our protein list to a GO analysis (Figure 6). All of the proteins were functionally categorized in 12 groups. In our dataset, the most enriched categories (Figure 6A) were biological process (35%) followed by molecular function (19%), Cellular component (16%), and others with (3%) each. The details of GO terms of those categories are described in (Figure 6B). As shown in the figure, some important annotations were reported in Cellular component category, such as localization, locomotion, and metabolism (Figure 6C).

#### PPIs and pathways

**Figure 7:**
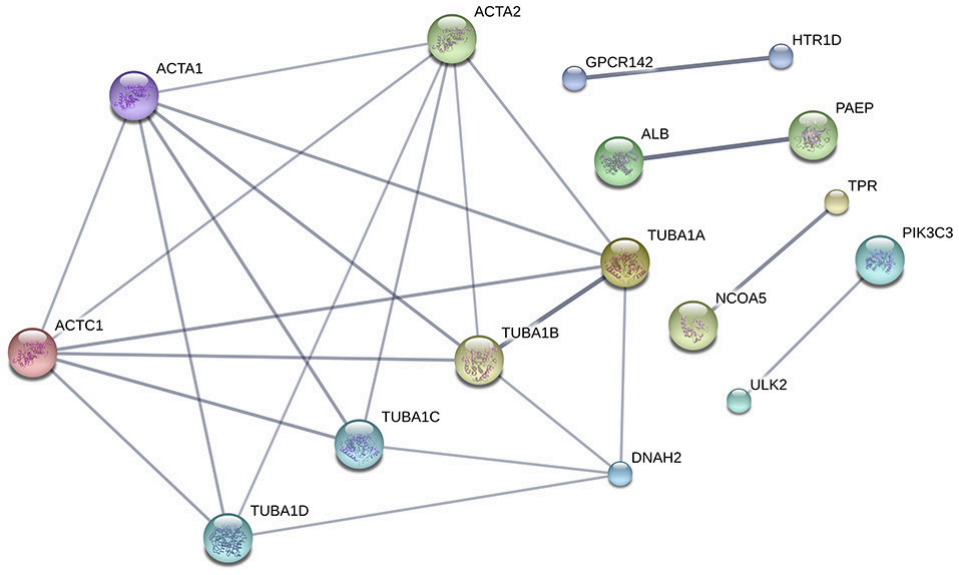
Protein-Protein Interaction [STRING-V10.0] (PPIs). The PPI was built using the following parameters: *Bos taurus* background (No match for *Bubalus bubalis* found, *B.bubalis* genome is not full and well-annotated yet). Highest confidence Score = 0.55. The dark line in the graph correspond to Highest confidence score higher than 0.7. Hide disconnected nodes in the network. PPI enrichment *p*-value = 0.000124, clustering coefficient: 0.902.

To summarize the proteomics results into a general functional model, we integrated known and predicted protein-protein interactions (PPIs) (Figure 7). The PPIs were built using STRING (Hu et al., 2008). We noted the presence of one network highlighted Tubulin-Tubulin and Actin-Tubulin interactions. In order to know more about their pathways we combined the STRING and PANTHER (Mi et al., 2016) pathway analyses. The list of relevant pathways (with at least two counts and *p* < 0.05) is described in Table 1. We observed several pathways confirming our previous finding. We noted that the tubulin, actin, and signaling pathways were enriched in our protein list. Those proteins are suggested to be implicated in spermatozoa acrosome assembly. This pathway analysis shows the presence of one molecular function, namely, the pathway concerning the structural constituent of the cytoskeleton. In fact, microfibrils are suggested to be the principal contractile organelles responsible for sperm motility (El Shoura, 1986).

**Table 1:** Pathways analysis (PANTHER-STRING) Count: Number of genes in the set.

**N°**	**PathwaysID**	**Pathway description**	**Source**	**Count**
1	**P00057**	Wnt signaling pathway	PANTHER-(v11)	3
2	**P00012**	Cadherin signaling pathway	PANTHER-(v11)	2
3	**P00031**	Inflammation mediated by chemokine and cytokine signaling pathway	PANTHER-(v11)	2
4	**P00044**	Nicotinic acetylcholine receptor signaling pathway	PANTHER-(v11)	2
5	**Kegg:04145**	Phagosome	KEGG(STRING,V10)	5
6	**Kegg:04540**	Gap junction	KEGG(STRING,V10)	4
7	IPR002452	Alpha tubulin	InterPro(STRING,V10)	3
8	IPR004001	Actin, conserved site	InterPro(STRING,V10)	3
9	IPR020902	Actin/actin-like conserved site	InterPro(STRING,V10)	3
10	IPR000217	Tubulin	InterPro(STRING,V10)	3
11	IPR003008	Tubulin/FtsZ, GTPase domain	InterPro(STRING,V10)	3
12	IPR003054	Keratin, type II	InterPro(STRING,V10)	3
13	IPR008280	Tubulin/FtsZ, C-terminal	InterPro(STRING,V10)	3
14	IPR017975	Tubulin, conserved site	InterPro(STRING,V10)	3
15	IPR018316	Tubulin/FtsZ, 2-layer sandwich domain	InterPro(STRING,V10)	3
16	IPR023123	Tubulin, C-terminal	InterPro(STRING,V10)	3
17	IPR004000	Actin family	InterPro(STRING,V10)	3
**BIOLOGICAL PROCESS**				
18	GO.0014902	Myotube differentiation	GO(STRING,V10)	3
**MOLECULAR FUNCTION**				
19	GO.0005200	Structural constituent of cytoskeleton	GO(STRING,V10)	3
**CELLULAR COMPONENT**				
20	GO.0005865	Striated muscle thin filament	GO(STRING,V10)	3
21	GO.0030017	Sarcomere	GO(STRING,V10)	4
22	GO.0001725	Stress fiber	GO(STRING,V10)	3
23	GO.0005884	Actin filament	GO(STRING,V10)	3
24	GO.0030016	Myofibril	GO(STRING,V10)	4
25	GO.0030175	Filopodium	GO(STRING,V10)	3
26	GO.0044430	Cytoskeletal part	GO(STRING,V10)	7
27	GO.0030027	Lamellipodium	GO(STRING,V10)	3

*The table shows pathways with significant value*.

#### PTM prediction

**Figure 8:**
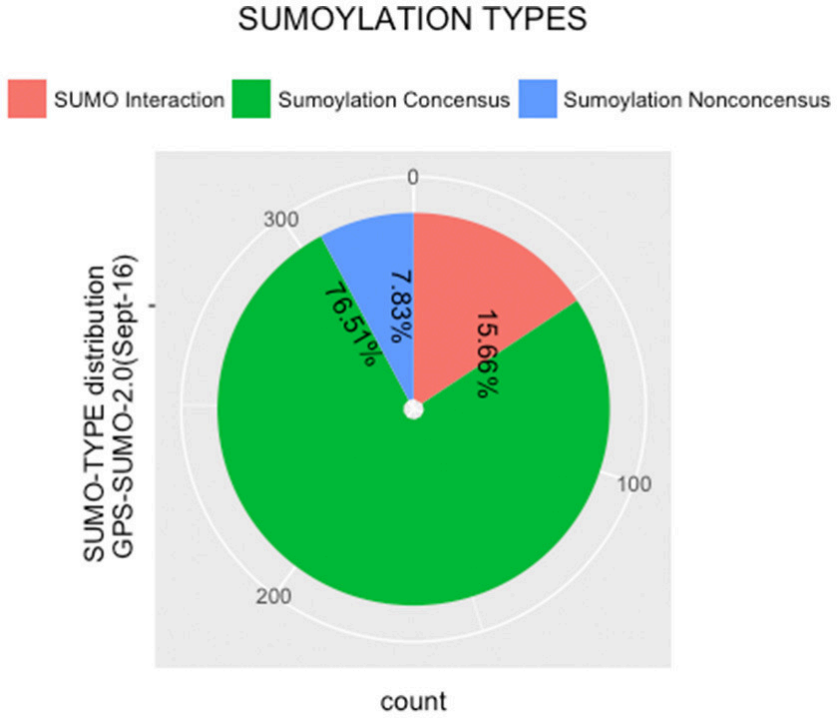
Distribution of SUMO modification types reported by (GPS-SUMO2.0).

**Figure 9:**
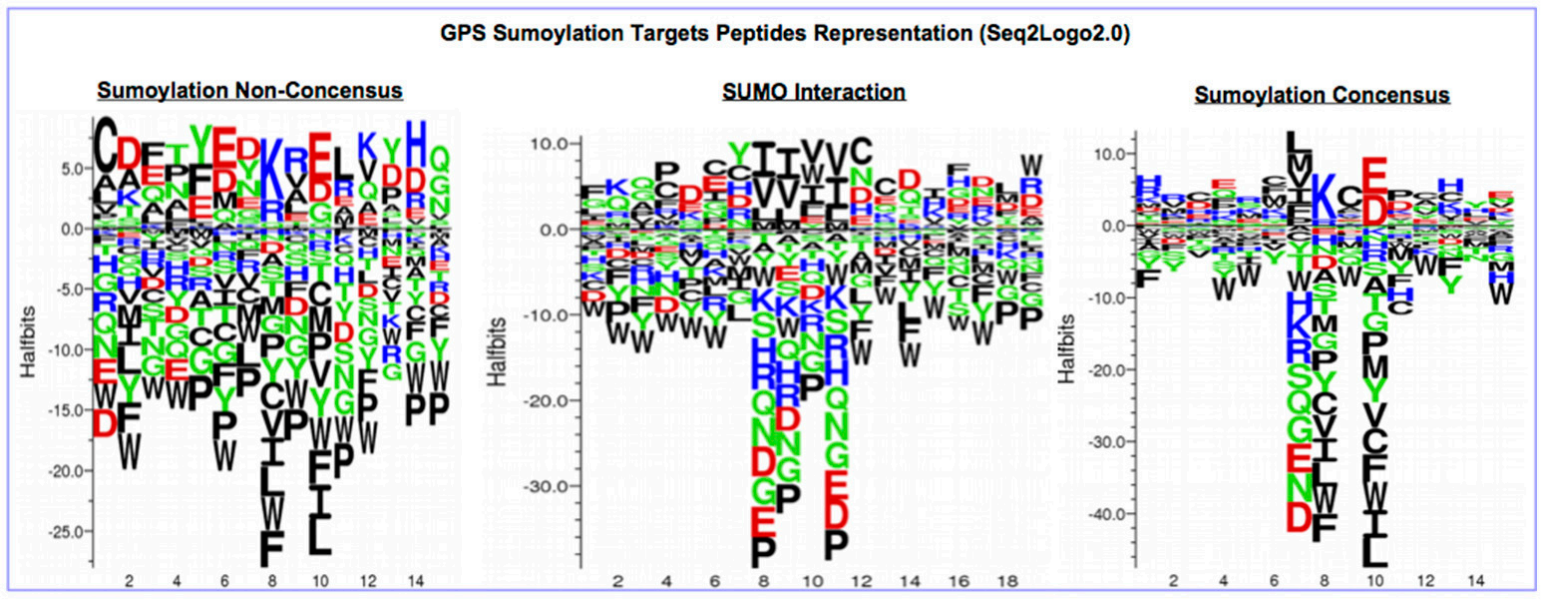
Seq2Logo2.0 representation of three types of SUMO modification peptides sequences reported by (GPS-SUMO2.0).

The protein sequences were scanned for potential SUMO targets. More than 400 in our identified list of proteins were reported by GPS-SUMO-v2.0 (Zhao et al., 2014) tool. We only kept 332 significant results (*p* < 0.05). The filtered results revealed three categories of SUMO modification (Figure 8). Only 7.8% of the proteins hold Sumoylation Non-Concensus (SNC) sites. About 15.6% of them present SUMO-Interaction (SI) sites. The majority of identified proteins (76.5%) contain Sumoylation Concensus (SC) sites. Representative peptide sequences were highlighted with Seq2Logo-v2.0 (Figure 9). Moreover, a deeper SUMO modification analysis revealed that some proteins might hold more than one SUMO modification sites type. In fact, one of them contains both SNC and SI sites, five of them contain both SNC and SC sites, eight present both SC and SI sites and three of them contain all types of SUMO modification sites (SI, SC, and SC). Twenty-two sequences hold only one type of SUMO modification site. The details are described in Table 2. In summary, we successfully predicted 46 potential SUMOylation targets between the 60 proteins identified by MS/MS. Based on 142 unique peptides (reported by the GPS-SUMO tool) analyzed, we predicted 10 Sumoylation Non-Concensus sites, 13 SUMO-interaction motifs, and 42 Sumoylation Concensus sites.

**Table 2:** Summary of bio-analysis findings SI, SUMO-Interaction; SNC, Sumoylation Non-Concensus; SC, Sumoylation Concensus. SCL (Ref. Table S2); COG categories (Ref. Figure 2); (^*^) (Ref. Discussion).

**GPS-SUMO-v2.0**				**JASSA-V4**			**iCell-package**	**EggNog**
**REF**.	**Protein name**	**Count**	**TypeGPS**	**Position**	**TypeJASSA**	**DB-Hits in UniProt**	**SCL**	**COG**
1	Zinc finger protein 668	2	SC	K56	Consensus inv	*Arabidopsis thaliana* [FLD (Q9CAE3)]	CT,N	K
1.1	Vinexin	1	SI	None	None	–	Cytoplasm	T
1.1	Vinexin	3	SC	K168	NDSM	*Homo sapiens* [TFAP2B (Q92481), TFAP2C (Q92754)]		
2	V(D)J recombination-activating protein 1	1	SC	K56	None	–	CT,N	L
2.1	Ubiquitin carboxyl-terminal hydrolase-48	1	SNC	K960	None	*Caenorhabditis elegans* [(Q19691)]	Cytoplasm	O
2.1	Ubiquitin carboxyl-terminal hydrolase-48	4	SC	K960	None	–		
2.2	Tumor necrosis factor alpha-induced protein 2	1	SNC	K663	None	–	Cytoplasm	U
2.2	Tumor necrosis factor alpha-induced protein 2	3	SC	K663	Extended PDSM	*Saccharomyces cerevisiae* [(P38904)]-Homo sapiens [(Q75MX6), (Q9UGLI), KLF3 (P57682), KLF8 (095600), SAFB2 (Q14151)]-Vaccinia virus [A40R(P21063)]		
G1	Tubulin alpha-1D chain-like	1	SC	K304	SUMO-Ac switch	–	Cytoplasm	A,Z
G2	Tubulin alpha-1C chain-like	1	SC	K230	SUMO-Ac switch	–	Cytoplasm	A,Z
G3	Tubulin alpha-1B chain	1	SC	K304	SUMO-Ac switch	–	Cytoplasm	A,Z
3	Tubulin alpha-1A chain-like	3	SC	K304	SUMO-Ac switch	–	CT,N	A,Z
4	Sterile alpha motif domain-containing protein 15	6	SC	K349	NDSM	Homo sapiens [RANBP2 (P49792), (Q15326)]	CT,N	S
5	Sperm-associated antigen 16 protein-like	1	SI	**No Match with JASSA**			CT,N	S,Z
6	SMAD4	2	SC	K159	NDSM	*Mus musculus* [(Q61624)]-Homo sapiens [SMAD4(Q13485), SOX6(P35712), SREBF2 (Q12772), ZNF148 (Q9UQR1)]-*Drosophila melanogaster* [(P09081-2), (Q7KPL1)]-*Saccharomyces cerevisiae* [(RPO21(P04050)]	CT,N	K,T
7	SMAD family member 4	2	SC	K159	NDSM	*Mus musculus* [(Q61624)]-Homo sapiens [SMAD4(Q13485), SOX6(P35712), SREBF2 (Q12772), ZNF148 (Q9UQR1)]-*Drosophila melanogaster* [(P09081-2), (Q7KPL1)]-*Saccharomyces cerevisiae* [(RPO21(P04050)]	Cytoplasm	K,KT
8	Serum albumin	3	SC	K204	HCSM	Homo sapiens [(Q12948), (P12004)]-*Drosophila melanogaster* [(Q7KPL1), *Arabidopsis thaliana* (Q9M7Q7)]	CT,EC	S,U,W
3.1	Serine/threonine-protein kinase	1	SI	AA 52-55	SIM Type β	Homo sapiens [Fir1 (P40020)]- *Saccharomyces cerevisiae* [Pc2/CBX4(000257)]	CT,N	T
Nek5								
3.1	Serine/threonine-protein kinase Nek5	1	SNC	K123	None	–		
3.1	Serine/threonine-protein kinase Nek5	3	SC	K123	SC-Direct	–		
9	Ribonuclease T2	1	SNC	**No Match with JASSA**			CT,EC	A
3.2	rho GTPase-activating protein 42-like	1	SC	K428	SC-Direct	*Rattus norvegians* [Ptk2 (035346)]-*Homo sapiens* [PTK2(Q05397)]	Cytoplasm	T
3.2	rho GTPase-activating protein 42-like	1	SI	AA 560-563	SIM Type β	*Saccharomyces cerevisiae* (Q06340)	Cytoplasm	
3.2	rho GTPase-activating protein 42-like	1	SNC	K428	SC-Inverted	*Drosophila melanogaster* [(P34021-3)]-*Trypanosoma cruzi* [(Q4DIR8)]	Cytoplasm	
10	Relaxin-3 receptor 2	1	SC	K272	SC-Direct	–	CM	T
11	Proteoglycan 3-like	1	SC	K300	SC-Direct	*Saccharomyces cerevisiae* [(P32526)]	Cytoplasm	TV
12	Protein FAM185A	2	SC	K300	SC-Direct	*Saccharomyces cerevisiae* [(P32526)]	Cytoplasm	S
13	Pro-neuregulin-2, membrane-bound isoforms	3	SC	K304	SC-Direct	*Homo sapiens* [(P05783)]	CM	S,T
14	Pregnancy-associated glycoprotein 6	2	SC	**No Match with JASSA**			CT,EC	O
12	Pregnancy-associated glycoprotein 11	1	SI	AA 13-16	SIM Type β	Homo sapiens [SETDB1 (Q15047)]	CT,EC	O
12	Pregnancy-associated glycoprotein 11	2	SC	–	–	–		
15	Pregnancy-associated glycoprotein 7	3	SC	**No Match with JASSA**			Cytoplasm	O
16	Phosphatidylinositol 3-kinase catalytic subunit type 3-like	5	SC	K68	SC-Direct	*Mus musculus* [(Q99PV8)]	CT,ED	T
17	Outer dense fiber protein 1	1	SC	K197	SC-Direct	*Mus musculus* [(Q99PV8)]	CT,N	Z
33	Nucleoprotein TPR	1	SNC	K585	SC-Inverted	*Saccharomyces cerevisiae* [MOTI (P32333)]	CT,N	*
33	Nucleoprotein TPR	3	SI	AA 456-462	SIM Type α	*Epstein-Barr virus* [(Q69140)]		
33	Nucleoprotein TPR	7	SC	K710	SC-Direct	*Homo sapiens* [(P05783)]		
13	Nuclear receptor coactivator 5	1	SC	AA 226-229	SIM Type α	Homo sapiens [Arkadia (Q6ZNA4)]	CT,N	K,UY
13	Nuclear receptor coactivator 5	1	SI	AA 202-205	SIM Type β	–		
18	Nuclear envelope pore membrane protein POM121C	1	SC	**No Match with JASSA**			CT,RE	S,U
14	NFX1-type zinc finger-containing protein 1	3	SI	AA 771-774	SIM Type α	Homo sapiens [Pc2/CBX4 (000257)]	Cytoplasm	L
14	NFX1-type zinc finger-containing protein 1	5	SC	K671	SC-Direct	Homo sapiens (095785)		
23	MDS1 and EVII complex locus protein EVII-like	1	SNC	K623	SC-Inverted	–	CT,N	K
23	MDS1 and EVII complex locus protein EVII-like	5	SC	K622	SC-Direct	–		
19	Latent-transforming growth factor beta-binding protein	1	SC	K362	SC-Direct	–	CT,EC	T
25	Keratin, type II cytoskeletal 5	1	SC		**No Match with JASSA**		Cytoplasm	S,T,Z
25	Keratin, type II cytoskeletal 5	1	SNC					
24	G patch domain-containing protein 4	1	SC	K138	SC-Direct	*Mus musculus* [(Q60864)]	CT,N	A,AD
24	G patch domain-containing protein 4	1	SNC	K200	SC-Inverted	–		
15	Filamin-C	1	SI	AA 413-416	SIM Type α	*Homo sapiens* [RNF4 (P78317)]	Cytoplasm	**
15	Filamin-C	6	SC	K894	SC-Direct	*Homo sapiens* [MORC3 (Q14149)]		
16	Fibrous sheath-interacting protein 2-like	3	SI	AA 906-909	SIM Type β	*Epstein-Barr virus* [(POC6Z8), BGLF4(P13288)]	CT,N	S
16	Fibrous sheath-interacting protein 2-like	16	SC	K212	SC-Direct	*M. musculus* [(P15806), Mitf (Q08874), Ncor1 (Q60974)]-*Homo sapiens* [ETV5 (P41161), *MITF (075030), TFE3 (P19532)]-S.cerevisiae [SGS1 (P35187)]*		
17	Dynein heavy chain 2, axonemal	3	SI	AA 4266-4269	SIM Type β	*Epstein-Barr virus* [(POC6Z8), BGLF4(P13288)]	CT,N,EC	Z
17	Dynein heavy chain 2, axonemal	7	SC	K4232	SC-Inverted	*Mus musculus [gsc* (Q02591)]		
41	Cadherin EGF LAG seven-pass G-type receptor 2	1	SNC	K2072	SC-Inverted	*A.thaliana* [AtHsfA2 (080982)]-*S.cerevisiae* [CDC3 (P32457)]	CM,CT	U,T,TW
41	Cadherin EGF LAG seven-pass G-type receptor 2	2	SI	AA 492-495	SIM Type 1	–		
18	Cadherin EGF LAG seven-pass G-type receptor 1	2	SC	AA 706-709	SIM Type 1	–	CM,CT	U,T,TW
18	Cadherin EGF LAG seven-pass G-type receptor 1	2	SI	AA 2339-2342	SIM Type α	*Salmo sala [Dgrn* (B5X9Z3)]		
20	BCL-6corepressor-like	2	SC	AA 447-450	SIM Type β	–	nucleus	K
21	ATP-dependent RNA helicase DDX54	1	SC	AA 696-699	SIM Type 1	*Epstein-Barr virus* (Q69138)	nucleus	A,O
22	ADP-ribosyltransferase 4	1	SC	K65	SC-Direct	*Homo sapiens* (P52565)	CM,CT,RE,MT	G
G2	Actin, gamma-enteric smooth muscle	1	SC	AA 249-251	SIM Type β	*Homo sapiens* [HIPK2 (Q9H2X6), Topor (Q9NS56)	Cytoplasm	Z
G2	Actin, aortic smooth muscle	1	SC	AA 249-252	SIM Type β	*Homo sapiens* [HIPK2 (Q9H2X6), Topor (Q9NS56)	Cytoplasm	Z
G2	Actin, alpha skeletal muscle	1	SC	AA 249-252	SIM Type β	*Homo sapiens* [HIPK2 (Q9H2X6), Topor (Q9NS56)	Cytoplasm	Z
G2	Actin, alpha cardiac muscle 1	1	SC	AA 249-252	SIM Type β	*Homo sapiens* [HIPK2 (Q9H2X6), Topor (Q9NS56)	Cytoplasm	Z

*REF, Reference in Discussion Section; PROTEIN NAME, Protein predicted as Sumoylated by GPS-SUMO; COUNT, Number of unique sequences; Type GPS, Type of Sumoylation reported by GPS-SUMO*.

*POSITION, K (Lysine)/Motif position; Dbhit, Several relevant Uniprot hits reported by JASSA*.

*Type JASSA, Type of Sumoylation predicted by JASSA (Please check the patterns list at http://www.jassa.fr, for further informations)*.

SI, SUMO-Interaction; SNC, Sumoylation Non-Concensus; SC, Sumoylation Concensus. SCL, (Ref. Table S2); COG categories (Ref. Figure 2); (^*^) (Ref. Discussion).

*JASSA was launched with the following parameters: Best predictions, Motifs: all types, high-cut-off [PSmax = 38.183 | Cut-off = 2.032]*.

*SIM, SUMO-interacting motifs (SIM)*.

*SUMO, putatifs SUMO site: Consensus direct/indirect*.

*NDSM, Negatively charged amino acid-dependent SUMOylation site*.

*PDSM, Phosphorylation-dependent SUMOylation motif*.

*HCSM, Hydrophobic cluster SUMOylation motif*.

*Psmax, Maximum Predictive Score; Ps, Predicted score direct; Ps, Predicted score Inverted; SC-Direct, Strong Consensus Direct; SC-Inverted, Strong Consensus Inverted*.

*White, Protein without isoforms in the list; Light green, Protein with 2 isoforms; Light Yellow, Protein with 3 isoforms; Light Blue, Group 1 and Group 2 mentioned in the text; Dark green, No COG annotation was founded for those proteins*.

Conscious about the validation of the potential sumoylated proteins, we submitted the *B. bubalis* protein sequences to the JASSA tool. We used this predictor not only to confirm the sumoylated motifs and sites previously identified by GPS-SUMO-V2.0, but also to determine whether those targets were revealed in other species. Thus, as presented in Table 2, about 90% of the targets identified by GPS-SUMO-V2.0 were confirmed by JASSA. Nevertheless, this tool shows that only 52% of the targets are present in mammals, 33% in Humans and 10% in mice. We noted that 13% were predicted in *Saccharomyces crvicae* and 6% in *Drosophila melanogaster*. Furthermore, 18% of the sumoylated potential sites present SUMO-Interacting motifs type SIM Type β (Song et al., 2004) and about 6% of these show the type SUMO-Acetyl switch (Stankovic-Valentin et al., 2007).

## Discussion

Sperm are essentially transcriptionally and translationally inactive. This implicates the PTM of proteins as essential in determining sperm morphology, motility, and functionality and, hence, in fundamentally influencing male fertility. To our knowledge, this is the first study of SUMO1 modification of proteins of buffalo spermatozoa. Thus, in this study, we addressed the role of sumoylation in Buffalo bull spermatozoa, an important PTM implicated in many cellular functions, such as DNA recombination, repair and replication, RNA transcription, nucleo-cytoplasmic trafficking, and protein stability. We confirmed that the expression of SUMO-1, was concentrated in the mid piece, neck, and head of the bull sperm and was lost upon induction of the sperm acrosome reaction with Ca^2+^ ionophore A23187. We also identified a suite of 60 bull sperm proteins that could potentially be modified by SUMO1, with implications for sperm functionality and male fertility. Importantly, 10 (17%) of these proteins were identified in other studies as sumoylated proteins, suggesting the likelihood that these are truly sumoylated proteins. This finding presents a great opportunity for future studies regarding validation of these findings and exploring the proteins in more detail.

Western blot analysis suggested that sperm capacitation induces changes in the sumoylation pattern in buffalo bull sperm. However, more sensitive techniques would be required to further dissect these differences, for example 2D gel at individual SUMO targets. This finding is in contrast to in other species, such as humans, where capacitation did not significantly alter the global level of sumoylation in sperm (Vigodner et al., 2013).

Spermatogenesis is a differentiation process with the end goal of functional sperm production, involving marked alterations in genetics, cell functions, and chromatin (Lena et al., 2016; Renata et al., 2016) Modification of sperm proteins by sumoylation, including those found in the mid piece, neck, head, and acrosomal region, therefore, could be important for many aspects of sperm function.

Sumoylation of sperm proteins at various stages of spermatogenesis was previously demonstrated in other species, including humans and rodents (Rogers et al., 2004; Vigodner and Morris, 2005; Vigodner et al., 2006, 2013; Brown et al., 2008; Marchiani et al., 2011, 2014; Stanton et al., 2012). However, the role of SUMO in spermatogenesis is as yet undefined, with involvement in processes, including heterochromatin organization, DNA repair mechanisms, and regulation of gene expression, all being suggested (Rogers et al., 2004; Vigodner et al., 2006; Stanton et al., 2012). In fact, the role of SUMO1 may be deleterious in some circumstances. While SUMO1 appears to be expressed in most mature human sperm (Marchiani et al., 2011, 2014), increased/excessive sumoylation is associated with an abnormal sperm morphology and motility (Vigodner et al., 2013; Marchiani et al., 2014) and with sperm DNA fragmentation (Marchiani et al., 2014).

In other cell types, SUMO has been shown to play a role in DNA repair. It is possible that, in sperm, SUMO may be involved an attempt to limit DNA damage or regulate mitochondrial dynamics, but in excess it may also have a deleterious effect on morphology and motility. The extent and the location of SUMO in sperm are likely to dictate the role sumoylation plays under different circumstances. Targets that have been identified in mature human sperm include the mitochondrial protein DRP1, the microtubule-organizing RanGAP1 and topoisomerase IIa, which is involved in chromatin remodeling (Marchiani et al., 2014).

We identified several proteins that are modified by SUMO1 in bull sperm. These include relaxin receptors, which have been recently associated with beneficial effects in human and boar spermatozoa motility and functionality (Ferlin et al., 2012; Feugang et al., 2015a). In boar spermatozoa, for example, relaxin and its receptors are distributed over the whole sperm length, and relaxin is particularly concentrated in the acrosome region (Feugang et al., 2015b). Our identification of the relaxin receptors RXFP4 relaxin(RLN) /INSL like family peptide 4, RLN-3/INSL7 receptor 2, and LOC102283812 RLN-3 receptor 1-like as potential SUMO1 targets in bull spermatozoa, therefore, has potential implications for sperm motility and functionality and, hence, for male fertility.

Remodeling of the cytoskeleton is also essential in sperm motility. We identified tubulin alpha-1C and alpha-1B chains, alpha and gamma actins ACTC1 and ACTG2, the axonemal protein DNAH2 dynein, and the intermediate filament components KRT4, KRT5, and KRT6B, among other cytoskeletal components, as potential SUMO1 targets in bull sperm. In un-reacted bull sperm, both actin and tubulin are shown to be localized in the equatorial segment of the acrosome actin, with actin also in the marginal acrosomal ridge of the heads of unreacted buffalo spermatozoa and tubulin around the acrosome periphery (Oikonomopoulou et al., 2009). This matches the localization we established for SUMO1 in buffalo sperm. Acrosomal expression of both actin and tubulin falls dramatically upon acrosome reaction induction (Oikonomopoulou et al., 2009), just as we identified for SUMO1 expression in buffalo sperm. The role of sumoylation in modification of cytoskeletal proteins, such as tubulin and actin, is not yet well-understood. In fact, tubulin itself is not well-recognized as a direct sumoylation target. Actually, the MAPs, including Pac1p, Bik1p, Kar9p, Tau, and Ndc80p, are more recognized targets (Alonso et al., 2015). However, we identified tubulin chains as potential direct targets of SUMO1 and suggest that this protein modification might play a role in regulating the induction of the acrosome reaction. Moreover, tubulin alpha-1C chain and ACTA1 were identified by others as sumoylated, and ACTA1 was specifically identified during mouse spermatogenesis (Becker et al., 2013; Xiao et al., 2016).

The sumoylation of actin, predominantly the nuclear form, was previously identified at position K284, and SUMO2 and SUMO3 were identified as the SUMO isoforms most likely to modify actin (Hofmann et al., 2009). However, we identified various actin types as potential SUMO1 targets in buffalo sperm. The potential effects of actin sumoylation include possible restriction of actin filament formation and the blocking of export of nuclear actin and the potential formation of alternative nuclear actin structures (Hofmann et al., 2009; Alonso et al., 2015), which again could contribute to regulating the acrosome reaction.

Sumoylation of the cytoplasmic dynein adaptor and target proteins were identified. However, no reports have yet described the direct SUMO-modulation of dynein (Alonso et al., 2015. Flagellar dynein is distinct from cytoplasmic dynein. Dynein is a molecular motor protein on the exterior doublet microtubules of sperm that generates energy for the momentum of flagella that is crucial for spermatozoa movement toward oocyte into female reproductive tract. Thus, sumoylation of the axonemal protein DNAH2 dynein has potential implications for male fertility. Meanwhile, sumoylation of the human keratin variants K8, K18, and K19 was demonstrated in hepatocytes, with implications for the organization of filaments and keratin solubility (Snider et al., 2011). More specifically, hyper sumoylation was associated with reduced keratin solubility and with chronic liver injury in both human and mouse livers. This hypersumoylation reduced the cytoprotective function of K8, K18, and K19 by retaining them in an insoluble compartment (Snider et al., 2011). Such effects on the spermatozoa keratins KRT4, KRT5, and KRT6B could therefore have a deleterious effect on sperm function.

Beyond cytoskeleton regulation, RAG1, which is essential for IgG immunoglobulin synthesis and class switching, was another potentially important SUMO1 targets identified in this study. IgG and RAG-1/RAG-2 expression was recently detected in human sperm, and an anti-IgG antibody was shown to inhibit sperm penetration of Zona-free hamster egg (Yan et al., 2016). Thus, sumoylation of RAG-1, which, to the best of our knowledge, has not previously reported, may be of importance in fertilization. In addition, SMAD4, a transcriptional regulator and key modulator of TGF-b signaling, was also identified as a SUMO1 target in buffalo sperm. The TGF-b signaling pathway was identified to be essential for maintaining adult spermatogenesis in mice (Moreno et al., 2010). The expression of this protein in the Sertoli and Leydig cells was recently identified as essential for the development of murine testes and testicular tumor suppression (Archambeault and Yao, 2014). The sumoylation of SMAD4 represses its transcriptional activity (Long et al., 2004). Thus, SMAD4 and its role in TGF-b signaling might be implicated in spermatogenesis in buffalo bull, and this pathway would be of interest to explore in the future. Other potentially important SUMO1 targets, in terms of bull fertility, include ODF1, which is involved in oocyte membrane fusion and SPAG16 (sperm associated antigen 16). ODF-1 was previously identified as a sumoylated protein human sperm (Vigodner et al., 2013). A deficiency in SPAG16 is associated with infertility in male mice due to impaired sperm motility (Zhang et al., 2006). The sumoylation of these two proteins might have affects on fertility in buffalo bulls.

It is common (but not always) that ubiquitination is associated with proteolytic pathways, and this is not the case of SUMO modification target protein, which are more implicated in PPIs, nuclear-cytoplasmic transport and the formation of specific domains (Vigodner and Morris, 2005).

To summarize our findings in Table 2, we detected 66 potential target of SUMO modification and we validated 59 of them within a second tool (JASSA). We highlighted the presence of two groups that were both predicted in the cytoplasm and present in the SC motifs. G2 is the actin protein group that is below the cytoskeleton COG category, and G1 is the tubulin protein group that is below to both the cytoskeleton and the RNA processing and modification COG categories. Furthermore, we observed an interesting motif type (SUMO-Acetyl switch) that is implicated in acetylation/deacetylation-SUMOylation switch (Stankovic-Valentin et al., 2007). This motif was detected exclusively in the G1 set of the tubulin proteins validated as SUMO targets (Table 2). Many studies (Wloga et al., 2010; Bhagwat et al., 2014) suggest that acetylated tubulin may affect sperm mobility. In addition, we noted that 18% of the predicted targets contain the SUMO-Interacting motifs type SIM Type β. This motif is suggested to be involved in the RanBP2/Nup358 and RanGAP1 interaction (Song et al., 2004). Those proteins seems to be very important for spermatozoa, since they are SUMO targets for RanGAP1 and Topoisomerase IIa in somatic and/or germ cells in mature human spermatozoa, as described in a previous study (Marchiani et al., 2014). We noted the presence of different kingdoms (plants, bacteria, virus) in the Uniport database hit match proposed by JASSA. These are known as Bovine Sperm YWHA Binding Partners (Puri et al., 2008).

**Figure 10:**
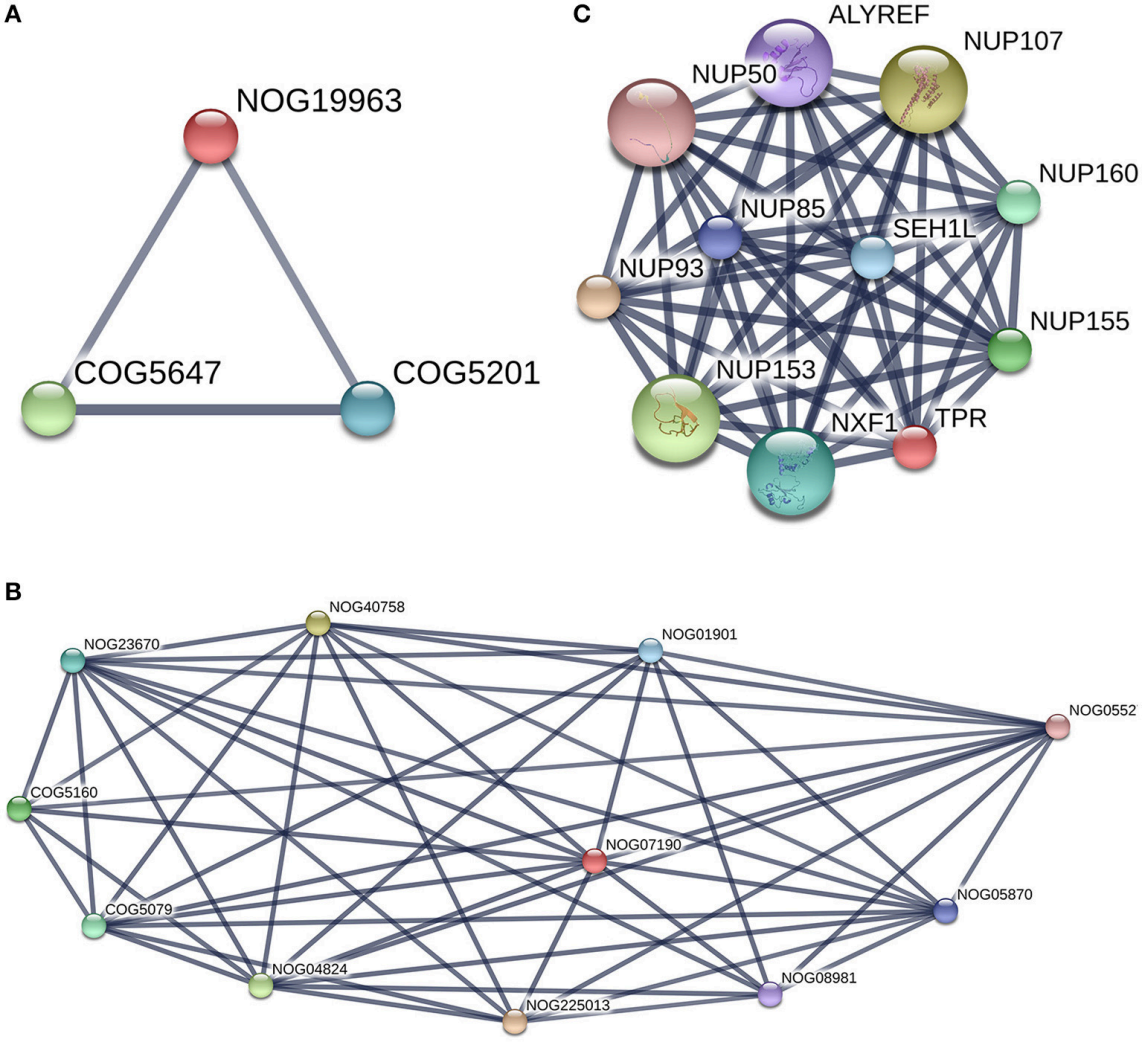
TPR and Filamin-C analysis with [STRING-V10.0]. Protein of interest is marked in Red in the graph. **(A)** TPR protein-protein interaction network. (Highest confidence Score = 0.9. Hide disconnected nodes in the network. PPI enrichment *p*-value = 0); **(B)** Filamin-C, COG analysis. This protein is marked as Non Supervised Orthologous Group (NOG19963) and is below to three edges of the graph with two COGs terms [COG5160 andCOG5079]. **(C)** COG analysis for TPR protein with string. TPR is marked as Non Supervised Orthologous Group (NOG07190). Two COGs terms are present in the graph [COG5646 andCOG5201].

PPIs between the two groups were observed (Figure 7). Furthermore, we do note two proteins without COG classification (marked with ^*^ in Table 2). The first one was TPR (XP_006060592.1), there was no match found in the EggNOG4.5 mammalian database for TPR. We reported the COG STRING results for the corresponding analysis in Figure 10C. TPR (NOG19963) seems to be connected to (COG5160: SUMO1 sentrin specific peptidase, Protease Ulp1 family which is below to [O: Post-translational modification, protein turnover, and chaperones] COG category, and COG5079: minichromosome maintenance complex component 3 associated protein and nuclear protein export factor, which is below to [DU:Cell cycle control, cell division, chromosome partitioning, Intracellular trafficking, secretion, and vesicular transport] COG category.

The details of the TPR PPIs (Figure 10A) and pathways reported by STRING are described in Table 3. The second protein reported in Table 3 without a COG prediction was Filamin-C (XP_006074441.x), and it was reported (for the 7 isomers) with EggNOG as being bellow the Z (cytoskeleton category) but with a score lower than 100. With the STRING tool, we observed that Filamin-C protein formed three nodes with two other COG terms (COG5647: cullin 1, a subunit of E3 ubiquitin ligase andCOG5201: ubiquitin-dependent protein catabolic process, SCF ubiquitin ligase - SKP1 component) (Figure 10B) that were both below the (O: Post-translational modification, protein turnover, and chaperones) COG category. There was no network reported with STRING for Filamin-C, it is an uncharacterized protein in the *bos* genus. A better annotation of *B. bubalis* genome may open the way for the use of other strategies (such as methods based on homology sequences analysis) to support our findings. In addition, studies using freshly collected sperm are need to validate these findings as the freeze-thaw process is known to induce sumoylation changes.

**Table 3:** Nucleoprotein TPR, pathways [STRING-v10] Count: count in gene set.

**No**	**Pathways ID**	**Pathway description**	**Source**	**COUNT**	**FDR**
**KEGG PATHWAYS**					
1	3013	RNA transport	KEGG(STRING,V10)	11	8.29E-022
**BIOLOGICAL PROCESS**					
2	GO.0051028	mRNA transport	GO(STRING,V10)	5	2.67E-008
3	GO.0006403	RNA localization	GO(STRING,V10)	5	3.12E-008
4	GO.0006999	Nuclear pore organization	GO(STRING,V10)	2	0.000325
5	GO.0006406	mRNA export from nucleus	GO(STRING,V10)	2	0.0142
6	GO.0071427	mRNA-containing ribonucleoprotein complex export from nucleus	GO(STRING,V10)	2	0.0142
7	GO.0071166	Ribonucleoprotein complex localization	GO(STRING,V10)	2	0.0247
**CELLULAR COMPONENT**					
8	GO.0005643	Nuclear pore	GO(STRING,V10)	3	1.77E-005
9	GO.0031080	Nuclear pore outer ring	GO(STRING,V10)	2	0.000493
10	GO.0005635	Nuclear envelope	GO(STRING,V10)	3	0.00527
11	GO.0000777	Condensed chromosome kinetochore	GO(STRING,V10)	2	0.0433
12	GO.0000779	Condensed chromosome, centromeric region	GO(STRING,V10)	2	0.0433

*FDR REPLACE, False Discovery Rate reported by STRING*.

In conclusion, we have confirmed that the expression of SUMO1 in sperm cells of buffalo bull is concentrated in the mid piece, neck, and head. We have identified a large number of SUMO1 targets which presents opportunities for future studies to contribute to a better understanding of the biological processes and pathways involved in spermatogenesis, the implications of sumoylation, including hyper sumoylation, for bull sperm function and male fertility.

## Author contributions

RB and LH conceived and designed the experiment; RB, LW, and JC performed experiments and interpreted the data; NH, ZR, CH, DW, DB, and HT contributed reagents/materials/analysis tools; RB and LH wrote and revised the paper, and all the authors approved the final version.

### Conflict of interest statement

The authors declare that the research was conducted in the absence of any commercial or financial relationships that could be construed as a potential conflict of interest.
