# Supplementary material for: Expression, Localization of SUMO-1, and Analyses of Potential SUMOylated Proteins in Bubalus bubalis Spermatozoa
Source: Front Physiol. 2017 Jun 13;8:354. doi: 10.3389/fphys.2017.00354 (PMC5468435; doi:10.3389/fphys.2017.00354)
Supplement: Supplementary file 1 [file DataSheet1.docx]

**Table S1 supplementary information**. Identification of SUMO-1 targets proteins unique to buffalo spermatozoa in the IP sample via Mass Spectrometry.

| **No** | **GI** | **Name** |
| --- | --- | --- |
| 1 | gi:122140942 | *Tubulin alpha-1C chain |
| 2 | gi:125912 | Beta-lactoglobulin or Beta-LG |
| 3 | gi: 223634674 | Tubulin alpha-1 B chain or tubulin alpha-ubiquitous chain |
| 4 | gi:258513337 | BCOR BCL6 corepressor |
| 5 | gi:258513337 | LOC787476 fibrous sheath-interacting protein 2-like |
| 6 | gi:258513338 | ZDHHC24 zinc finger, DHHC-type containing 24 |
| 7 | gi:258513343 | *PIK3C3 phosphatidylinositol 3-kinase,catalytic subunit type 3 |
| 8 | gi:258513344 | LTA lymphotoxin alpha |
| 9 | gi:258513352 | ARHGAP42 Rho GTPase activating protein 42 |
| 10 | gi:258513352 | RAG1 recombination activating gene 1 |
| 11 | gi:258513356 | PAEP progestagen-associated endometrial protein |
| 12 | gi:258513356 | LTBP1 latent transforming growth factor beta binding protein 1 |
| 13 | gi:258513359 | SORBS3 sorbin and SH3 domain containing 3 |
| 14 | gi:258513360 | NRG2 neuregulin 2 |
| 15 | gi:258513362 | CELSR1 cadherin, EGF LAG seven-pass G-type receptor 1 |
| 16 | gi:258513362 | KRT4 keratin 4 |
| 17 | gi:258513362 | KRT6B keratin 6B |
| 18 | gi:258513362 | STYK1 srine/threonine/tyrosine kinase 1 |
| 19 | gi:258513363 | FAM185A family with sequence similarity 185, member A |
| 20 | gi:258513364 | RXFP4 relaxin/insulin-like family peptide receptor 4 |
| 21 | gi:258513365 | USP48 ubiquitin specific peptidase 48 |
| 22 | gi:355477164 | *AHNAK AHNAK nucleoprotein |
| 23 | gi:355477172 | TNFAIP2 tumor necrosis factor, alpha induced protein 2 |
| 24 | gi:355477183 | SAMD15 sterile alpha motif domain containing 15 |
| 25 | gi:417523480 | LOC101113573 proteoglycan 3-like |
| 26 | gi:417531886 | *ACTA1 actin, alpha 1, skeletal muscle |
| 27 | gi:417531888 | POM121C POM121 transmembrane nucleoporin C |
| 28 | gi:417531888 | *ZNF668 zinc finger protein 668 |
| 29 | gi:417531892 | ACTA2 actin, alpha 2, smooth muscle, aorta |
| 30 | gi:417531919 | DNAH2 dynein, axonemal, heavy chain 2 |
| 31 | gi:417531923 | *L0C101107110 serine/threonine-protein kinase Nek5 |
| 32 | gi:417531923 | ERICH6B glutamate-rich 6B |
| 33 | gi:417531943 | ACTC1 actin. Alpha, cardiac muscle 1 |
| 34 | gi:417531959 | *FLNC filamin C, gamma |
| 35 | gi:417531961 | KRT5 keratin 5, type II |
| 36 | gi:417531962 | SPAG16 sperm associated antigen 16 |
| 37 | gi:417531962 | HTR1D 5-hydroxytryptamine (serotonin) receptor 1D, G protein-coupled |
| 38 | gi:417531964 | OMA1 OMA1 zinc metallopeptidase |
| 39 | gi:417531964 | GPATCH4 G patch domain containing 4 |
| 40 | gi:541128973 | *ODF1 outer dense fiber of sperm tails 1 |
| 41 | gi:541128978 | LOC102174742 uncharacterized |
| 42 | gi:550706067 | DPH2 DPH2 homolog (S. cerevisiae) |
| 43 | gi:550706258 | LOC102283812 relaxin-3 receptor 1-like |
| 44 | gi:550707187 | *DDX54 DEAD (Asp-Glu-Ala-Asp) box polypeptide 54 |
| 45 | gi:550709826 | RXFP4 relaxin/insulin-like family peptide receptor 4 |
| 46 | gi:550710310 | ACTG2 actin, gamma 2, smooth muscle, enteric |
| 47 | gi:550710365 | NCOA5 anuclear receptor coactivator 5 |
| 48 | gi:550710581 | *SMAD4 SMAD family member 4 |
| 49 | gi:550710641 | LOC102270390 tubulin alpha-1A chain-like |
| 50 | gi:550711326 | LOC102277557 EGF-like module-containing mucin-like hormone receptor-like 3-like |
| 51 | gi:550711462 | LOC102281213 tubulin alpha-1D chain-like |
| 52 | gi:550711526 | PNTPT1 polyribonucleotide nucleotidyltransferase 1 |
| 53 | gi:550711778 | ULK2 unc-51 like autophagy activating kinase 2 |
| 54 | gi:586830479 | Pregnancy-associated glycoprotein 6; ovPAG6 |
| 55 | gi:75071141 | Relaxin-3/INSL7 receptor 2 |
| 56 | gi:75074838 | Pregnancy-associated glycoprontein-11 |
| 57 | gi:75074841 | Pregnancy-associated glycoprontein-7 |
| 58 | gi:761983806 | MECOM MDS1 and EVI1 complex locus |
| 59 | gi:761984074 | ART4 ADP-ribosyltransferase 4 (Dombrock blood group) |
| 60 | gi:761984316 | ALB albumin |

Note: Protein with red asterisk (*) were identified in previous studies as being sumoylated.

**Table S2 supplementary Information:** SCL reported by the iLoc-Animal and iLoc-Euk tools _A ([Sub Cellular Location] reported by iLoc-Animal tool), E ([Sub Cellular Location] reported by iLoc-Euk tool, GO: Gene Ontology, PSSM:Position Specific-Scoring Matrix), NR(SCL not reported neither by iLoc-Animal or iLoc-Euk), NA(No result Available for this sequence). **C:** cytoplasm**, EC:** endoplasmic, **R**: Reticulum, **M:** mitochondrion, **CM:** cell membrane, **EC:** extracellular, N**:** nucleus, **ED:** endosome.

Note:

A ([Sub Cellular Location] reported by iLoc-Animal tool), E ([Sub Cellular Location] reported by iLoc-Euk tool).

Methods Used by the tool (GO: Gene Ontology, PSSM: Position Specific-Scoring Matrix).

NR (SCL no location found for the sequence neither by iLoc-Animal or iLoc-Euk).

NA (No result Available for this sequence).
